# A simple approach to identifying community and healthcare locations for hand hygiene interventions during public health emergencies – Uganda, 2020–2022

**DOI:** 10.1186/s12982-026-02802-4

**Published:** 2026-10-01

**Authors:** Alexandra Medley, Kanako Ishida, Sauda Yapswale, Francis Ocitti, Maureen Kesande, Fred Tusabe, Victoria Trinies, Christiana Hug, Caroline Pratt, Christy Craig, Mohammed Lamorde, David Berendes

**Affiliations:** 1 Division of Foodborne, Waterborne, and Environmental Diseases, National Center for Emerging and Zoonotic Infectious Diseases, Centers for Disease Control and Prevention, Atlanta, GA USA; 2 Infectious Disease Institute, Makerere University, Kampala, Uganda; 3 Oak Ridge Institute for Science and Education, Oak Ridge, TN USA

## Abstract

**Background:**

As part of Uganda’s COVID-19 emergency response, two districts along high-volume trucker routes linked to early clusters, Amuru and Tororo, were prioritized for hand hygiene (HH) interventions. While HH is a critical non-pharmaceutical intervention, tools to efficiently identify high-risk community locations during outbreaks are limited.

**Methods:**

We adapted the Population Connectivity Across Borders (PopCAB) tool, developed by the U.S. Centers for Disease Control and Prevention, to identify and prioritize high-risk community locations and healthcare facilities (HCF) and inform targeted HH interventions, including provision of alcohol-based hand rub (ABHR), handwashing stations (HWS), and site-level support. Additional questions assessed HH infrastructure and ABHR perceptions. A mixed-methods baseline assessment was followed by implementation and post-intervention assessments. Quantitative indicators were compared using chi-square tests. Qualitative data were analyzed using deductive thematic analysis.

**Results:**

Between 2020 and 2021, four focus group discussions and 17 key informant interviews with 56 participants identified 734 locations. Fifty-nine community sites and 21 HCF were prioritized. At baseline, HWS availability varied, and community members often preferred ABHR over shared stations. Between baseline and follow-up, HWS and ABHR availability in community locations increased from 63 to 85% and 40 to 83%, respectively. In HCF, sufficient ABHR availability increased from 9 to 91%. However, consistent access was constrained by theft, breakage, supply shortages, and limited maintenance systems.

**Conclusion:**

PopCAB identified high-risk locations often overlooked by routine WASH programming and informed targeted HH interventions. While access improved, sustainability challenges highlight the need for strengthened operational and maintenance strategies in emergency response settings.

**Supplementary Information:**

The online version contains supplementary material available at https://doi.org/10.1186/s12982-026-02802-4.

## Introduction

The emergence of SARS-CoV-2 and the resulting COVID-19 pandemic presented unprecedented challenges to health systems globally, particularly in low- and middle-income countries (LMIC) with limited access to vaccines and other pharmaceutical interventions [1]. In Uganda, the first confirmed case of COVID-19 disease was reported on March 21 of 2020 [2]. Over the next few months, domestic spread of COVID-19 occurred primarily in the long-distance truck driver (LDTD) population, and this group was strongly linked to further community spread [3, 4]. Truck drivers performed an essential role in maintaining supply chains and services in communities and thus were likely to interact with people in highly frequented community locations such as points of entry (POE), markets, guesthouses, places of worship, and schools, as well as healthcare facilities (HCF) [5]. In these types of community locations and HCF in LMIC, especially those with limited access to vaccination or other pharmaceutical interventions, consistent and adequate access to non-pharmaceutical measures are critical to reduce disease transmission, especially of respiratory diseases like COVID-19 [6].

Hand hygiene (HH), defined as handwashing with soap and water or using alcohol-based hand rub (ABHR), is a key prevention measure recommended to reduce the disease burden worldwide, especially during the COVID-19 pandemic [7–9]. ABHR and handwashing stations (HWS) with water and soap are two important HH resources, but their availability is often limited in community locations of LMIC, as well as in clinical and non-clinical areas within HCF [6, 10, 11]. Lack of HH resources risks personal exposure to infections; the World Health Organization (WHO) interim Global Water, Sanitation and Hygiene (WASH) guidance during the COVID-19 pandemic stated that frequent and correct HH was one of the most important measures to prevent infection with SARS-CoV-2 [12]. Access to commercially produced ABHR in LMIC during public health emergencies like the COVID-19 pandemic is often limited and extremely costly [13, 14]. In community settings specifically, HH resources are generally not available to many community members due to cost inflation, high demand, supply chain issues, challenges in maintaining and sustaining access, and the perceived impression that critical HH materials are most needed in healthcare settings [6].

Prior to the pandemic, the Infectious Diseases Institute (IDI), in partnership with the US Centers for Disease Control and Prevention (CDC) had begun local production of ABHR for HCF in a small number of predominantly rural districts of Uganda [15–17]. This local production and distribution model was extended to include high-risk community locations during the pandemic, because whilst healthcare settings critically require these materials, focusing solely on healthcare settings without consideration of other types of settings may leave community members challenged to perform adequate HH in settings where the risk of transmission could be high [18]. In emergency response settings, the rapid identification of priority community and healthcare settings where these interventions are critically needed remains an elusive and often ad hoc task, especially as most HH evaluations have been limited to households, schools, and HCF [19]. Given the need to prioritize HWS and ABHR access in settings of greatest need, IDI and CDC sought to identify locations for HH interventions where LDTD might contribute significantly to the spread of COVID-19, and evaluate sustainability of WASH interventions such as local ABHR production, HWS and ABHR provision, and basic management engagement, in these locations.

To identify priority community locations and HCF in two border districts, IDI used the Population Connectivity Across Borders (PopCAB) package, which is a low-burden tool used to characterize the formal and informal movement patterns and connectivity of mobile populations across and around borders [20]. PopCAB facilitates the collection of qualitative, quantitative, and geospatial data through national- and local-level focus group discussions (FGD) or key informant interviews (KII) with participatory mapping [21–23]. This toolkit has been implemented in multiple African countries to address a variety of needs, including overall preparedness planning to inform responses to Ebola and Lassa fever outbreaks and to map the mobility of animals to prevent the spread of zoonotic diseases [22, 33]. This toolkit was developed to be adaptable to different scenarios, especially outbreak scenarios. IDI adapted the toolkit in coordination with CDC to identify participants who would have knowledge of LDTD movement cross-border and within Uganda, with the purpose of identifying community locations and HCF that may be at higher risk of contact with this mobile group.

The objectives were to adapt and implement PopCAB for identifying priority community settings and HCF at higher risk for COVID-19 and other infectious disease transmission due to their connection to the highly mobile LDTD population; understand HH attitudes and practices of people who use these community locations, especially their challenges to access and perform HH; assess how well the adapted tool can inform interventions in novel (community or publicly-accessible areas at HCF) settings; and assess the availability of HH materials in PopCAB-prioritized community locations, before and after interventions focused on HH supply position and management. Results from this assessment can inform decision making for identifying and supporting HH infrastructure at HCF and prioritized community locations in resource-limited settings, especially in the context of acute disease epidemics.

## Methods

### Population

As part of emergency response to the COVID-19 pandemic, two districts were selected—Amuru, which borders South Sudan to the north, and Tororo, which borders Kenya to the east—due to their locations along high-volume trucker routes, of concern as contributors to early clusters of COVID-19 [3]. Both districts have large POE with immigration and customs clearing offices and thus a high volume of travelers and LDTD entering and exiting Uganda.

### Activity overview and timeline

PopCAB implementation to identify priority HH intervention sites took place in November 2020. Baseline assessment of HH services in prioritized community and HCF locations began in January 2021, with interventions implemented 1–2 months after baseline. Follow-up assessments were conducted approximately 6 months after baseline. Details of each phase of the activity are provided below.

### PopCAB facilitated location identification and hand hygiene perceptions

We adapted the PopCAB toolkit, which takes a mixed-methods approach, to identify community locations and HCF that may be at higher risk for contact with LDTD (Supplement 1). KII were used to guide a participatory mapping exercise to identify locations and routes, as well as information about the locations. Key topics of the FGD and KII discussion guides included characteristics of travelers who travel into/through/out of the area; why these people visit the area; where people come from when they visit the area and where they go after they visit; mode of transport to reach the area; and how long people stay. We also developed additional FGD and KII questions to inform development of interventions that were tailored to the context, including questions about HH needs, infrastructure, general perceptions of ABHR, and how HH infrastructure setups and HH ownership, responsibility, and management in community locations operate and function. We used ArcGIS Desktop version 10.3.1 (Esri, Redlands, CA, USA) to create basemaps for Amuru and Tororo districts. Administrative boundaries were pulled from the Global Administrative Areas database (GADM), and additional shapefiles such as HCF, markets, and POE were downloaded from OpenStreetMap through the Geofabrik website [25–27]. We applied consistent symbology to represent different types of facilities, including POE, hospitals, and health centers, as well as administrative levels such as district, sub-county, and parish. To make the maps easier to read during fieldwork, we simplified the labels and used strong visual contrast. Each map included a north arrow, scale bar, legend, and labeled administrative areas to help ensure that PopCAB respondents could interpret them easily. Both maps included the southern portion of South Sudan (Amuru) and western Kenya (Tororo) given the focus on cross-border movement.

Initial PopCAB KII and FGD were conducted with the highest-level respondent type (here, the District Health Office). Additional stakeholders were identified through snowball sampling, where information from each round of interviews was used to identify increasingly granular levels of stakeholders and conduct subsequent KII/FGD until enough information was obtained to identify priority locations in need of more HH resources. Prior to the FGD or KII, participants were oriented to the purpose of the activity and given time to become familiar and comfortable with the map. Facilitators from Uganda’s Ministry of Health (MoH) and IDI recorded verbatim transcripts of the FGD and KII. Facilitators and notetakers collaborated to link specific locations and routes on the map with the verbatim notes using unique letter IDs. After each FGD and KII, the notes were consolidated, unique IDs checked, and map markings clarified for correctness. Using the typed notes and annotated map, each unique location and route was geocoded with latitude and longitude, and key variables were entered into a Microsoft Excel (Microsoft Corporation, Redmond, WA, USA) database. Each KII or FGD required two or three facilitators and took approximately one hour to conduct, and another hour to enter and validate data in the field.

Based on the anticipated cost of the interventions, IDI estimated between 60 and 80 locations could be prioritized for interventions. IDI and the District Health Office originally selected 25 priority locations in Amuru and 35 priority locations in Tororo: locations included POE, roadside checkpoints within the district, guesthouses, markets, schools, places of worship, police stations, and HCF. Factors used to prioritize included perceived increased risk of COVID-19 transmission at the location by local health officials, volume of people at the location, and frequency of visitors. Immediately after baseline HH services assessments were conducted by IDI at these 60 locations, a secondary FGD was conducted with the Uganda Truck Drivers Association in which IDI identified an additional 20 priority locations for HH services assessments and interventions, and assessments were promptly conducted by IDI as part of the baseline.

### Assessment of prioritized locations

Project staff conducted quantitative assessments of HH services in the selected locations at two time points: baseline assessments conducted immediately prior to implementing HH interventions, and follow-up assessments conducted at available/accessible locations approximately 6 months after the baseline assessment and establishment of the ABHR production unit and distribution system in each district was completed.

Both baseline and follow-up assessments included a quantitative HH service assessment tool that included a survey with the facility manager to assess the number of people (at community locations) or admissions and outpatients (at HCF) entering the premises and water sources, as well as direct observation of the presence and condition of ABHR and handwashing resources. Minor changes to improve the facilitator experience were made to the HH service assessment tool after the baseline assessments were complete, based on facilitator feedback. HH service assessment data were collected using mobile data collection platforms, and SurveyCTO, an Open Data Kit (ODK)-based mobile electronic data collection platform, for baseline and Kobo Toolbox, which is also an ODK-based mobile electronic data collection platform., for follow-up (ODK Inc, San Diego, CA; Dobility, Inc., Cambridge, MA, USA; KoboToolbox, Cambridge, MA, USA).

To better understand the specific HH service needs at community locations, which are less uniform and predictable than HCF, we also worked with managers of each community location at baseline to develop schematic maps of each location. The maps included where HH stations were present and key points where they might be required, such as entrances, toilets, and eating areas. Although HCF entrances were assessed for HH stations as they are publicly accessible locations, HH stations in clinical areas of HCF were not assessed or captured as part of this assessment. These maps not only were used to assess current resources, but also provided information on resource gaps that would inform interventions. At follow-up, additional qualitative KII were conducted with managers in a convenience sample of community locations. KII guides included questions on perceptions of the interventions and successes and challenges with management and maintenance of HH stations. Interviews were conducted either by phone or in person and were recorded and transcribed.

### Interventions

Following the baseline assessment, we implemented HH services interventions including: working with the District Health Office to identify sites for ABHR production, training on ABHR production, producing and distributing ABHR to all identified priority locations based on person-volume, providing HWS at key locations such as latrines, providing education on HH and use of these resources to focal persons (such as site managers) at sites, and monitoring ABHR stock and resupplying as needed. Education resources for community members on HH were abundant due to other COVID-19 related education measures, and we did not supply additional such resources.

### Analytic approach

Quantitative HH service assessment data were analyzed descriptively using SAS version 9.4 (SAS Institute, Cary, NC). Analyses were restricted to locations with both baseline and follow-up data available, as follow-up data collection was not feasible in some locations because of COVID-19–related disruptions, regional insecurity, and other operational constraints. Similarly, we selected and analyzed variables that were present across both baseline and follow-up datasets to adequately compare them. The statistical significance of changes from baseline to follow-up were assessed using Pearson’s chi-squared test and a p<0.05 threshold. Qualitative interviews were transcribed and analyzed using a deductive analysis approach. An a priori codebook was developed based on the interview guide and included an open code to account for emerging themes in the data. Interview text falling under the codes was extracted into Excel, following which the extracted text within each code was reviewed to identify themes.

## Results

### PopCAB facilitated location identification

We conducted 2 FGD, with 10 participants each, and 9 KII, for a total of 29 participants) in Amuru district, and 2 FGD, with 19 participants total, and 8 KII, for a total of 27 participants in Tororo district. Participants represented: district health, infection prevention and control, and surveillance staff; POE immigration, border health screeners, Uganda revenue authority, and border internal security officers; village health teams; district police and security officers; truck drivers; and lodging owners.

**Fig. 1:**
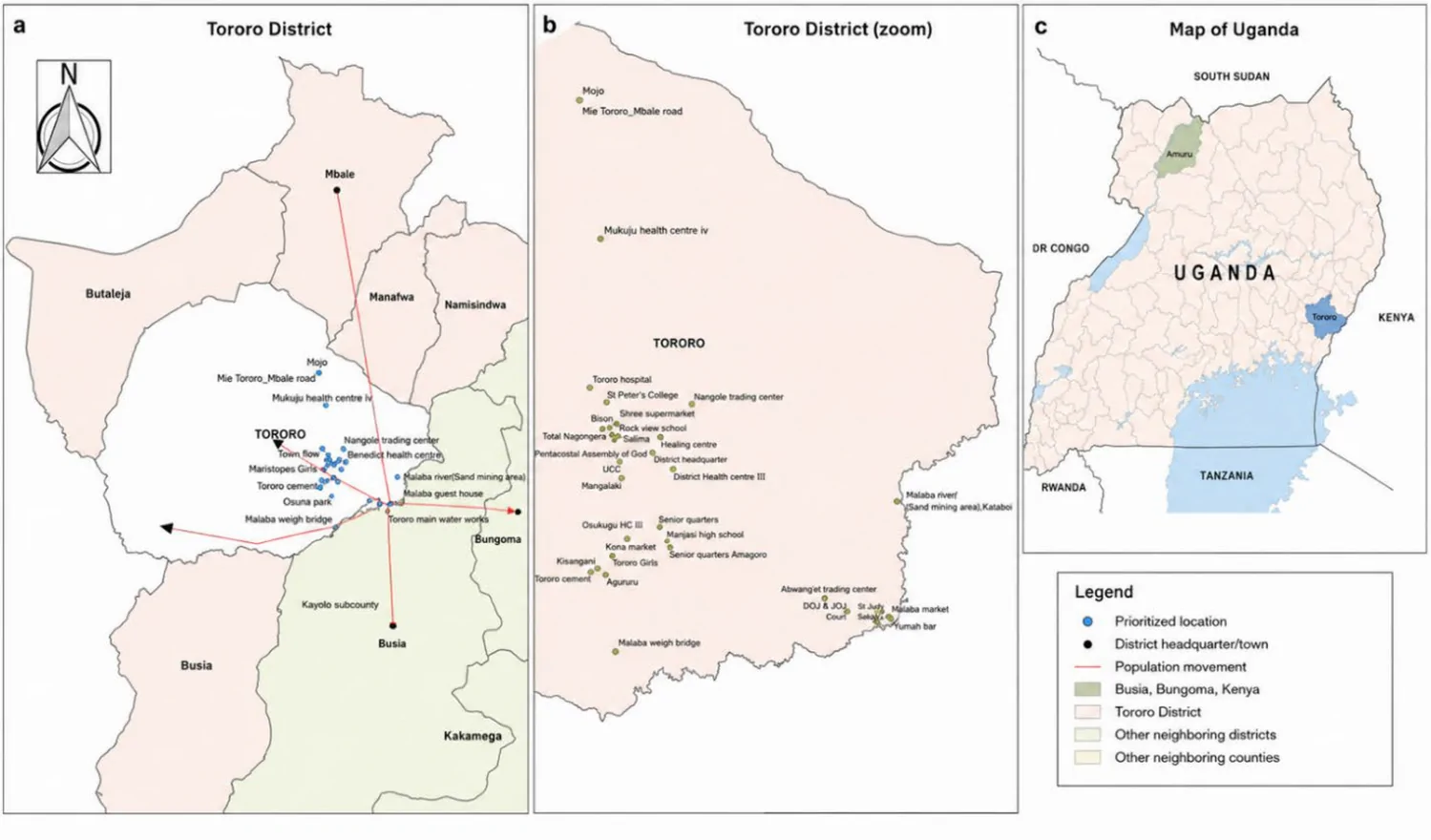
Maps of study districts and prioritized locations: **a** Tororo District showing prioritized locations and population movement patterns; **b** Tororo district highlighting prioritized sites; **c** Map of Uganda showing Amuru and Tororo Districts

A total of 319 and 415 locations where LDTD might contribute significantly to the spread of COVID-19 were mentioned in Amuru and Tororo, respectively (Table 1). This list likely included duplicates, as locations could be listed in multiple KII and FGD with slight variations in name. Locations noted most frequently were grouped into the following categories: HCF, checkpoints, refugee reception points, schools and universities, guesthouses and lodging, POE, places of worship, and markets. Other locations such as factories, towns, police stations, town councils, municipalities, and sub-districts were mentioned with less frequency and were grouped together as “other” locations. Maps were created to illustrate the geographic spread and interconnectedness of higher priority locations for both districts (Fig. 1a–c). Ultimately, based on these findings and additional discussions with the Uganda Truck Drivers Association and public health officials, 80 locations were prioritized for HH service assessment and interventions.

**Table 1:** Community and healthcare facility locations identified with the Population Connectivity Across Borders tool and further prioritized for assessment and interventions

Location type and district	Total locations identified	Prioritized locations round 1^a^	Prioritized locations round 2^b,c^	Prioritized locations included for analysis^d^
All community locations	661	48	*59*	40
Tororo community locations	380	27	*30*	21
POE^e^ and checkpoints	138	8	*9*	3
Police Station	124	0	2	1
Guest houses	40	5	5	4
Markets	34	6	6	5
Schools	31	4	4	4
Place of worship	13	4	4	4
Amuru Community Locations	281	21	*29*	19
POE and checkpoints	100	7	*12*	5
Police station	87	0	*3*	0
Markets	44	4	4	4
Schools	26	3	3	3
Guest houses	21	7	7	7
Place of worship	3	0	0	0
Healthcare Facilities (HCF)	73	12	*21*	12
Tororo HCF	35	8	*13*	8
Amuru HCF	38	4	*8*	4
All locations	734	60	80	52

^a^Removal of duplicates, locations with no known GPS identifiers, and a final list prioritized based on the PopCAB activity and discussion with district public health officers

^b^Additional priority locations included after further discussion with the Uganda Truck Drivers Association and public health officials

^**c**^Number of locations is italicized if the number of locations increased in round 2

^d^Locations that completed a baseline assessment, had interventions, and completed a follow-up assessment

^e^Points of entry

### PopCAB hand hygiene services assessments

Across both districts, most PopCAB FGD and KII participants said that they tried to practice handwashing to improve cleanliness, avoid the coronavirus, and prevent other infectious diseases or avoid germs. Participants stated that they performed HH before or after eating food, after using the toilet, after interacting with many people, or touching surfaces or documents touched by others. Participants reported that they tried to use soap and water when handwashing and some mentioned using ABHR when available.

Reported availability of HWS in different locations was varied. Some participants reported that stations were available in locations such as offices, schools, HCF entrances or toilets, supermarkets, and hotels; others said they were not. In addition, even when locations did have a station, participants noted that handwashing was frequently not possible due to lack of water or soap, insufficient number of stations, or broken stations.

Others reported that they did not like using HWS because they did not want to touch taps of shared stations due to the possibility of germs on them. Some participants said they understood general principles of handwashing but didn’t know the official steps for handwashing and that they or others likely did not follow the proper steps. A few participants reported the presence of informational materials on HH or posted rules regarding HH at the location, while most did not.

In general, participants said that ABHR was more convenient, quicker to use, and more user friendly than water and soap. However, ABHR was not used as often as people would like because they could not find it, afford it, or offices where they worked had run out of supplies. Participants reported different approaches to applying ABHR, including varying time spent rubbing their hands or amount applied.

### Assessment of prioritized locations

Of a total of 37 locations in Amuru and 43 locations in Tororo where interventions were implemented, we conducted both baseline and follow-up HH service assessments in 19 community locations and 4 HCF in Amuru and 21 community locations and 8 HCF in Tororo (Table 1).

**Fig. 2:**
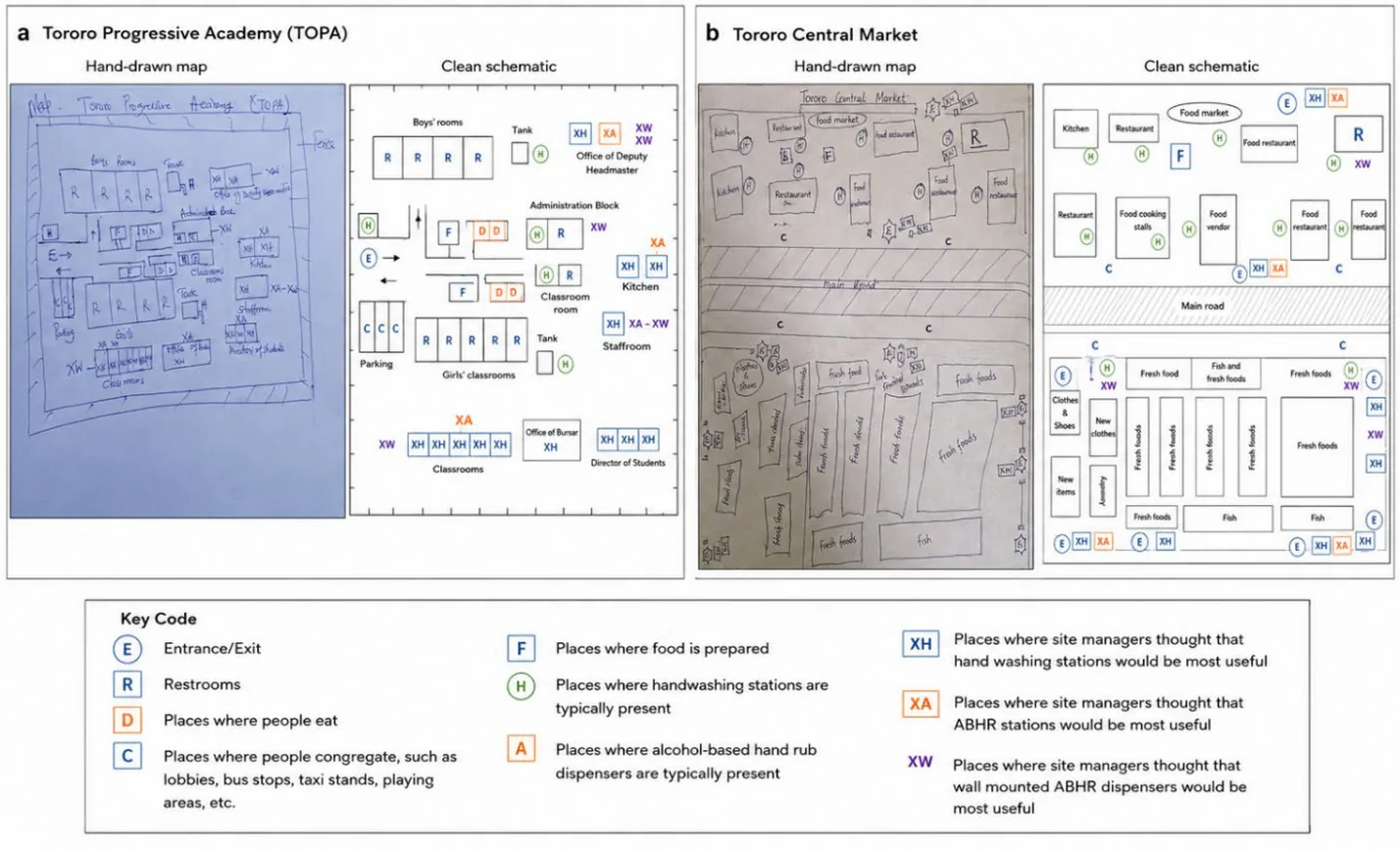
Examples of community location schematic maps used to identify placement and gaps in hand hygiene resources: **a** Tororo Progressive Academy; **b** Tororo Central Market

Prior to interventions, the most common water source for handwashing was piped water for both community locations (38%, n = 15) and HCF (58%, n = 7) (Table 2). In community locations, 15% (n = 6) did not provide handwashing facilities. Water sources were mostly located on the grounds of the locations (68%, n = 27 for community locations and 83%, n = 10 for HCF). Hand-drawn schematic maps of each location were created at baseline, and existing and desired handwashing stations identified with a keycode (Fig. 2a, b).

**Table 2:** Characteristics of locations prioritized for hand hygiene services (HH) baseline and follow-up assessments and HH interventions

Characteristics	Community		Healthcare Facilities	
	N = 40		N = 12	
District				
Amuru	19	48%	4	33%
Tororo	21	53%	8	67%
Location Type				
Guest Houses	11	28%	–	–
Market	9	23%	–	–
POE and Checkpoints^b^	8	20%	–	–
School	7	18%	–	–
Places of Worship	4	10%	–	–
Police Station	1	3%		
HCF Level^a^				
HC II	–	–	0	0%
HC III	–	–	3	25%
HC IV	–	–	6	50%
Hospital	–	–	3	25%
Average number of patrons and staff per day (mean, range)	676	(14–3020)	126	(22–417)
Main Water Source Used for Handwashing^c^				
Piped water	15	38%	7	58%
Borehole/Protected Dug Well	12	30%	5	42%
Unprotected Dug Well	7	18%	0	0%
Did not provide HW Facilities	6	15%	0	0%
Location of water source				
On the grounds of the location	27	68%	10	83%
Within 500 m	7	18%	2	17%
More than 500 m	0	0%	0	0%
Missing	6	15%	0	0%

^a^HCF within the Uganda healthcare system are classified into levels based on their catchment areas and the services they provide. The levels of HCF include Health Center (HC) II, which provides community-based, outpatient service; HC III, which additionally includes simple diagnostic and maternal health services; HC IV, which further includes surgical services; and hospital, which has specialized services in addition to all other services provided by lower-level HCF [28]. All levels of HCF were included in the assessment

^b^Points of entry, checkpoints, refugee reception centers

^c^These data come from the follow-up assessment

The pre- and post-intervention assessments contextualize how PopCAB-identified sites differed from locations typically prioritized for WASH programming and illustrate the operational realities of implementing HH services interventions in community settings during an emergency. The percentage of community locations with ABHR supplies significantly increased from 40% at baseline to 83% (p-value <0.05) at follow-up (Table 3). The main source of ABHR in community locations at baseline was through purchase (63%). At follow-up, the primary source of ABHR changed to IDI, the implementer of the intervention (100%); ABHR from either the government or other private organizations was additionally received by 12% of community locations. In HCF, ABHR access was 92% both at baseline and follow-up. At baseline, 73% of HCF reported that ABHR for HCF was provided by the government and a private organization other than IDI. At follow-up, 100% of HCF received ABHR from IDI; 45% of HCF continued to receive ABHR from the government and 36% from other private organizations.

**Table 3:** Hand hygiene (HH) materials availability reported during baseline and follow-up HH service assessments

	Community				Significance^a^	Healthcare Facilities				Significance^1^
	Baseline		Follow-up			Baseline		Follow-up		
	Jan-21		Aug-22			Jan-21		Aug-22		
	N = 40		N = 40			N = 12		N = 12		
ABHR										
Total Locations with ABHR Present	16	40%	33	83%	*	11	92%	11	92%	-
Source of ABHR^b^										
IDI	0	0%	33	100%	–	0	0%	11	100%	–
Other	10	63%	4	12%		11	100%	7	58%	
Purchase	10	63%	0	0%	–	2	18%	0	0%	–
Government	0	0%	1	3%	–	8	73%	5	45%	
Other Private Organization^c^	0	0%	3	9%	–	8	73%	4	36%	
Missing	6	38%	0	0%	–	0	0%	0	0%	–
Locations had sufficient ABHR in the last six months (baseline) / three months (follow-up)	4	25%	31	93%	***	1	9%	10	91%	*
Are there ever interruptions to the formal ABHR supply line?^2^										
Yes	8	50%	3	9%	-	4	36%	1	14%	
No	7	44%	30	91%	***	4	36%	10	91%	
Missing	1	6%	0	0%	–	3	27%	0	0%	-
Handwashing and water supply										
Location provides handwashing stations	25	63%	34	85%		–	–	–	–	–
Water supply for handwashing is sometimes interrupted	18	72%	20	59%		6	50%	5	42%	
Median number of months per year water is interrupted (range)	3	(1–14)	3	(1–8)		2	(0–6)	3	(1–11)	
There has been sufficient water for handwashing in the past two weeks	21	84%	28	82%		8	67%	9	75%	

^a^Pearson’s chi-squared test; values that did not change between time points or were missing or zero at either time point were not assessed

^b^Values may not total 100% as multiple supply sources could be reported

^c^Other organizations included World Vision, European Union, and Uganda Cares

^*^Significant at p<.05

^**^Significant at p<.01

^***^Significant at p<.001

The percentage of community locations reporting having enough ABHR significantly increased from 25 to 93% (p < 0.001) and the proportion of HCF increased from 9 to 91% (p < 0.05). At follow-up, community locations reporting no interruption to the ABHR supply line increased significantly from 44% at baseline to 91% at follow up (p < 0.001). Although water interruption was common among locations providing HWS (72% at baseline and 59% at follow-up at community locations and 50% and 42% at HCF), the majority of locations with HWS reported having enough water for handwashing (84% at baseline and 82% at follow-up at community locations and 67% and 75% at HCF). Presence of HWS in community locations increased from 63% at baseline to 85% at follow-up; we collected data on the presence of HWS at entrances for HCF.

### Post-intervention key informant follow-up interviews

In follow-up KII in community locations, managers reported that the provision of ABHR and handwashing supplies facilitated HH in their locations. Some managers reported a preference for having ABHR in their locations due to ease of use. However, most said that HWS should be available as well, because they felt that some visitors were not familiar with how to use ABHR and reported people avoiding ABHR due to religious or personal beliefs about using alcohol. Managers generally expressed a preference for providing liquid soap at HWS due to bar soap being frequently stolen and user concerns that bar soap may be contaminated due to being frequently touched. Managers reported challenges with breakage and theft of HWS containers and taps, stations running out of soap, water, and ABHR during the day, and challenges with water supply in general. Several approaches were employed to overcome these challenges, including establishing designated responsibility for keeping facilities stocked during the day, constructing cages to prevent theft of soap and ABHR containers, and placing HWS and ABHR containers near guards or other designated people to monitor their use and condition throughout the day.

## Discussion and conclusion

We modified PopCAB methodology to assess HH resources and attitudes in locations frequented by LDTD to support mitigation efforts in Uganda early in the COVID-19 pandemic. Adapting PopCAB methods for identifying locations that attract a large volume of mobile populations and LDTD with additional HH service assessments was efficient and identified high-risk community locations that would not typically be included in HH interventions. Qualitative investigation as part of the PopCAB methodology, specifically, yielded unique insights into gaps in knowledge on use and management of particular HH resources that were critical to improve HH practices. Assessments of HH services infrastructure revealed that water access for HH and alternatives, such as ABHR, were low in community locations, which informed the interventions used.

PopCAB has supported responses to multiple public health emergencies, including COVID-19, across Africa by identifying high-risk mobility patterns, a key driver of communicable disease spread [29]. PopCAB was also implemented in the US to characterize farmer mobility at the US-Mexico border for vaccination prioritization [30]. Additionally, PopCAB has supported public health events or preparedness such as informing risk communication strategies and vaccination guidelines for yellow fever and identifying cross-border animal movement for zoonotic disease risk identification [23, 24].

Our adaptation uniquely extended its application to efficiently assess underrepresented community settings in Uganda using limited resources to combine population movement data with HH service assessments to identify and prioritize key community locations and HCF for interventions during the COVID-19 pandemic. This unique ability to assess risks from population movement alongside gaps in HH services was necessary to comprehensively respond to the need for access across community locations [31, 32]. Many of these locations, including border crossings and other mass gathering locations in communities, may not have been prioritized for resources based on traditional HH services monitoring, which has focused, outside of households, on schools and HCF alone [19].

There were some challenges implementing this method, including accounting for duplication of locations during data analysis, as well as the potential for bias from respondents with regard to locations of most interest to them and their community, potentially at the cost of other priority locations [33]. Location prioritization bias could be mitigated through a more systematic selection of respondents to improve representativeness. Follow-up meetings, including those with the Uganda Truck Drivers Association and with the District Health Offices, allowed for other locations to be added to improve the representativeness of the final prioritized list that was agreed on by public health officials. This combined population movement/HH service assessment approach can be an effective first step in responding to other outbreaks or epidemics of respiratory diseases where HH must be prioritized as a mitigation measure.

The mixed-methods PopCAB approach enabled investigators to not only identify priority locations but also obtain a baseline knowledge of the perceived needs and challenges with supporting HH in community locations. Additionally, the resources and time required to conduct PopCAB were relatively modest, with only 6 facilitators required to conduct 21 FGD and KII over 5 days to identify 734 potential sites for interventions, making it a practical approach for rapid, community-informed assessment. This was critical information, given they were novel locations for HH services interventions for whom HH supplies (HWS and ABHR), communications, and educational needs were not well understood [34]. For example, the information gathered in the FGD and KII indicated that although there was fairly high awareness and acceptability of ABHR, people did not have reliable access to it in these locations and nor did they know when and how to use it correctly. In our broader evaluation, we found that integration of ABHR and HWS and education on appropriate HH practices in community locations could rapidly improve HH [35]. Discussions with location managers during baseline assessments also enabled selection of preferred attributes of ABHR dispensers, including modifications to reduce theft. Given that most standardized HH services or WASH assessments based on the Joint Monitoring Program are exclusively survey-based, qualitative assessment can yield novel insights into immediate and short-term gaps in behavioral aspects of HH, such as use and maintenance, that are crucial to the success of response efforts [34, 36–39].

Qualitative assessments were also critical to informing ongoing challenges with further investigation or applied research. Findings from the KII conducted with managers at follow-up revealed challenges with management of HWS in less monitored/supervised locations and additional details on user preferences, including a desire for HWS in addition to ABHR. Managers reported issues with maintaining the stations, including lacking standard operating procedures for cleaning and disinfection and resources for preventing or repairing damage. Future interventions in community locations could consider incorporating location managers and supervisors at both evaluation and intervention phases, as their engagement is likely critical to meeting the needs specific to each location given the heterogeneity of community location infrastructures (including sustainable, local access to water for handwashing) and the diverse needs or preferences of the populations who frequent them [19].

In community settings, post-intervention availability reflects functional access at the time of assessment rather than an expectation of continuous or universal coverage, which is rarely feasible given diffuse ownership, infrastructure and resource constraints, and maintenance challenges. Our quantitative assessments revealed that access to water and ABHR were both larger limitations for community locations compared to HCF, which informed approaches to response measures. Availability of handwashing stations in community sites was sub-optimal (63–85%), indicating that continued provision of ABHR may fill a significant gap in the immediate term. However, although ABHR can be a temporary solution to address immediate needs, it may not be an appropriate long-term substitute given that location managers felt HWS should also be available to provide HH options for people who are unfamiliar with ABHR or unwilling to use it due to personal or religious reasons. Access to ABHR prior to the intervention was low: less than half of community locations had ABHR and less than a quarter had enough to cover all their needs despite the perceived need for access during the height of COVID-19 when concern was high and interventions by other donors or government could have been concurrently implemented (and thus, more ABHR could be expected to be present at the baseline evaluation). After the intervention, community locations with ABHR present doubled, and almost all had enough ABHR to cover their needs, mostly due to a local ABHR production program that facilitated both HCF and new community locations-specific access [38]. Although HH services access in HCF was better than in community locations, the establishment of the local ABHR program increased access to a sufficient amount of ABHR from 9 to 91% and reduced reliance on other sources of ABHR, suggesting that continued access to locally-produced ABHR could enable more sustained access to HH services in HCF where HH programs are already in place [13, 16, 39].

There are a few limitations in this assessment. First, due to challenges accessing some community locations, our analysis only included 52 locations where both a baseline and a post-intervention follow-up assessment were conducted; thus, our findings may not fully represent the 80 prioritized locations. Second, there may have been other similar interventions in these locations that influenced our results. Finally, small differences in how enumerators evaluated, including due to minor improvements to assessment tools between baseline and follow-up could have introduced procedural bias.

### Conclusion

PopCAB identified high-risk locations that are often overlooked by routine WASH monitoring and programming frameworks. These insights guided targeted interventions that improved HH services access in high-mobility settings, although maintenance challenges with infrastructure and supply chains remained, indicating that sustainability is challenging. Though not commonly used in WASH, PopCAB shows promise for outbreak-related community-level HH strategies. We observed that HH services interventions, including local ABHR production, were able to improve access to HH resources, particularly in water-scarce areas in community locations. However, renewed focus on HH resource management is required to sustain behavior change and ensure continued HH services access and HH performance.

## Supplementary Information

Additional file 1.

## Data Availability

The data that support the findings of this study are available from the corresponding author upon reasonable request.
