# Supplementary material for: A simple approach to identifying community and healthcare locations for hand hygiene interventions during public health emergencies – Uganda, 2020–2022
Source: Discov Public Health. 2026 Oct 1;23(1):1564. doi: 10.1186/s12982-026-02802-4 (PMC13630945; doi:10.1186/s12982-026-02802-4)
Supplement: Supplementary file 1 [file 12982_2026_2802_MOESM1_ESM.docx]

**Supplement 1:** **Population Connectivity Across Borders (PopCAB) Facilitator Guide**

Focus group discussion guide for facilitators

Original template developed by the CDC Global Border Health Team in September 2016

Adapted by IDI for HH and ABHR Interventions in October 2020

Document purpose: To provide general questions to facilitate the collection of qualitative information on domestic and cross-border human mobility patterns to inform more targeted public health interventions. Additional probing questions are provided to catalyze discussion towards the targeted narrative.

Introduction provided by the facilitator

- Organization(s) affiliated with activity
- Additional staff assisting or observing the discussion
- Activity purpose: To gather their perspectives on what types of people move or travel and why they move or travel to various points of interest and to use

this general information to strengthen health programs.

- Ethical considerations

o Voluntary and anonymous participation with no names recorded and individual level

information not shared

o Ask permission to take photos or record session
- Orient participants to the map
  o Describe, and seek participation in, identifying cardinal directions and points of reference

o Describe that the group will use the map to strengthen the discussion by identifying points or routes of interest directly on the map

Introduction provided by the participants

- Each participant, as interested, should describe their background as it relates to the design of the focus group discussion.
- For example, if the facilitator is talking with a group of traders,

the participants can talk about where and what they trade.

If the facilitator gathered a group of people of one religion, the participants could describe where they are from and to which religious congregation site they belong.

| **Map Orientation Activity** | | |
| --- | --- | --- |
| **Questions** | **Notes** | **Purpose** |
| 1. Please identify and describe, using the map, areas of interest you visit in this general area?  *Probes:*  i. When you travel for personal reasons where do you go? Why?  ii. Where are you from or where were you born? How often do you visit your home village/town and why?  iii. Where does your family reside? How often do you visit them or they visit you?  iv. Where do you often buy/sell produce? Or livestock? | i. Location and route descriptions can be general, e.g. to sell goods, for health reasons, visit family, etc. More information will be collected throughout the discussion.  ii. Include towns, highways, health facilities, markets, places of worship, schools, etc.  iii. Inform participants that the locations do not need to be confined to short distance travel or areas shown on the map. They should include locations they travel to near and far for any reason, including international locations. | *Gather introductory information on participants’ geographical sense of community and the areas they often visit, including where they live, if relevant. This list will aid in prioritizing points and routes of interest to discuss in subsequent questions.* |
| 2. Which of these identified areas of interest do you feel are also important for others in [*insert focus group* *genre: e.g. your profession, your tribe, your community*] and/or [*depending on the genre*] the groups you provide services to.  i. Why are these areas more important? | i. Encourage participants to discuss each area identified in question 1 to discuss if and why the sites are important for others in their community including others in their focus group genre or the groups to which they provide services.  ii. Aim to document on the map all the identified areas and transit points along travel routes. For all identified sites, collect GPS information after the focus group discussion event. | *Gather information on the level of importance of the identified areas of interest for the general public and/or the groups to which they provide services to aid in prioritizing a limited number of areas on which to focus during subsequent questions. These FGD are to inform IDI about where to distribute locally made ABHR, including HCF, POE, trucker routes and congregation points/rest stops. Depending on the participant group, ensure that these locations are prioritized in the following sections.* |

**Before proceeding, review all the sites and areas identified and, depending on available time, select 4 sites for further discussion.**

*Notes for this section:*

i. Question order is not critical as long as ‘who’, ‘why’, ‘to/from where’, ‘when’, and ‘how people travel’ are discussed for each area (ensure that probing questions about WASH and ABHR take place earlier in the FGD so that participants are still engaged – these are key questions).

ii. Continue to document information provided through the discussion for related locations and routes.

*Purpose of this section:*

Gather detailed mobility and connectivity information for a limited number of ‘priority’ identified areas or routes to develop a narrative about how identified locations are integrated with and connected to each other and the surrounding communities.

| **Follow up Questions for the 4 selected Sites for further Discussion** | |
| --- | --- |
| **Questions** | |
| 3. **Who:** Please describe who visits this area.    a. Discuss characteristics including age, profession, cultural identity, residential area including areas in bordering countries, etc.    b. Do new people come often or do the same people routinely visit? | |
| 4. **Why:** Describe why people visit this area.    a. Discuss reasons including but not limited to:       i. health care seeking (for specific illnesses or treatment?)       ii. selling/buying goods (what types of goods?)       iii. religious considerations (festivals or routine practice?)       iv. professional interests (markets, livelihood opportunities)       v. education (boarding schools, primary schools, etc.)       vi. long distance migration, family connections.    b. Additional probing questions:       vii. Are the services better or cheaper in this area of interest?       viii. Is travel to this area easier than to another area with similar services?       ix. Where appropriate, do rules or regulations, such as travel restrictions, seasonal trends, etc, result in more travel to this place? | |
| 5. **Hand hygiene**: “*Now, we would like to ask you questions about hand hygiene, which is important for us as we identify ways to improve access to and use of hand cleaning and sanitizing products.”*  a. Do you typically wash your hands with soap and water when you travel or are at this location?  IF YES:  i. Why do you wash your hands when traveling to or are at this location?  ii. When and where do you wash your hands? How often do you wash your hands? What do you use to wash your hands?  iii. *[If you use soap and water,]* do you use also alcohol-based hand sanitizer after washing your hands?  iv. Do you find the handwashing stations easy to use? Why or why not?  IF NO:  iii. Why do you not typically wash your hands when you travel to or are at this location? Is there anything that makes handwashing difficult when traveling to or at this location? If so, please describe.  iv. Do you use ABHR instead of washing hands with soap and water?  iv. If yes, can you describe to me how you typically apply alcohol-based hand sanitizer, including the amount used and duration of time spent rubbing your hands?  v. If no, what do you think are the barriers to using alcohol-based hand sanitizer?  b. Can you describe for me locations where sanitizers are commonly used?  i. Are there hand hygiene posters in the above locations? If yes, how many/what are the recommended steps for proper hand hygiene?  ii. If you work at any of these locations, are there rules in place about how often to perform hand hygiene and how to perform it?  iii. If yes, are there typically the supplies needed to follow the rules? (e.g. water/soap, ABHR, clear guidance, time to do so).  c. *[For truckers/drivers:]* Do you perform hand hygiene at POE, security stops, or when your goods are check (customs, etc.)?  i. If yes, ask the same questions about hand washing and ABHR (sections 5a and 5b)  ii. If there are others that travel with you, do they perform any type of hand hygiene? | |
| 6. **To/From where**: Describe where people who visit or live in this area are coming from when they visit / live in this area. Describe where they go after they visit or live in the area.  a. Facilitate a discussion to cover as much detail about areas of origin, down to village level or specific population demographics. If the origin area is urban, ask for specific neighborhoods or communities within the urban setting.  b. Ensure a discussion about where the people go after visiting the specific location, e.g. return home, pass on to an urban area, take purchased goods to a different market, etc. | |
| 7. **How**: Describe how people reach the area.   a. Describe what type of transportation people take to reach the location, e.g. train, rental car, bicycle, by foot, etc., and if this mode of transport is available throughout the year or in specific seasons, festivals, etc. b. If related travel occurs to/from a neighboring country, ask for information about common official and unofficial border crossings or routes. For all identified border crossings:    i. Is the border crossing point or route official or unofficial?    ii. Do people use different crossings or routes to leave the country then they did to enter it? If yes, why? E.g. do they use official crossings to enter the country and unofficial crossings to leave or vice versa    iii. From where do people come to cross the border through that location?    iv. Is this border crossing used for other purposes such as seeking health care or livelihood? | *Note*: This discussion may highlight a transit point commonly used to change transportation method that was unidentified in previous discussion. If so, gather more information about the transit point. |
| 8. **Duration**: Describe how long people stay when they visit this area, e.g. a day, a few hours, a few days, months?   c. Describe the daily, weekly, seasonal patterns for visiting or traveling through this area. d. For how long do people stay when they visit, e.g. a day, a few hours, a few days, months? e. For areas with an event, e.g. markets, festivals, do people come a few days or more before and    - or stay a few days or more after the event? f. Where do they stay overnight if they come from another community, e.g. hotels, residential areas, non-permanent structures, etc. | |
